# Evaluation of the risk of malnutrition in hospitalized children by PYMS, STAMP, and STRONGkids tools and comparison with their anthropometric indices: a cross-sectional study

**DOI:** 10.1186/s40795-022-00525-8

**Published:** 2022-04-21

**Authors:** Armen Malekiantaghi, Kosar AsnaAshari, Hosein Shabani-Mirzaee, Mohsen Vigeh, Mohsen Sadatinezhad, Kambiz Eftekhari

**Affiliations:** 1 Pediatric department, Pediatric Gastroenterology, Bahrami Children Hospital, Tehran University of Medical Sciences, Tehran, Iran; 2 Pediatric department, Bahrami Children Hospital, Tehran University of Medical Sciences, Tehran, Iran; 3 Pediatric department, Pediatric Endocrinology and Metabolism, Bahrami Children Hospital, Tehran University of Medical Sciences, Tehran, Iran; 4 Maternal-Fetal Medicine Research Center, Tehran University of Medical Sciences, Tehran, Iran; 5 Pediatric department, Pediatric Gastroenterology and Hepatology Research Center, Bahrami Children Hospital, Tehran University of Medical Sciences, Kiaee Street, 1641744991 Tehran, Iran

**Keywords:** Children, Malnutrition, STAMP, PYMS, STRONGkids

## Abstract

**Background:**

Malnutrition is a determining factor of pediatric mortality and morbidity, especially in low and middle-income countries. Hospitalized children are at a higher risk of malnutrition. Several malnutrition screening tools have been used, among which STAMP, PYMS, and STRONGkids are valid tools with high sensitivity and specificity. The aim of this study was to compare these screening tools to find the best ones in identifying the risk of malnutrition in hospitalized children.

**Methods:**

This is a cross-sectional study performed on hospitalized children aged 1 to 16 years. The questionnaires of PYMS, STAMP, STRONGkids malnutrition risk assessment tools were filled. The weight for height and BMI for age Z-scores were calculated. The data were analyzed by SPSS. Sensitivity, specificity, positive predictive value, and negative predictive values of the risk scores based on weight for height and BMI for age Z-scores were calculated.

**Results:**

Ninety-three patients with a mean age of 5.53 ± 3.9 years were included. The frequency of malnutrition was reported as 26% and 39% according to weight for height and BMI for age Z-scores, respectively. A significant relationship was found between PYMS and Weight for height Z-score (*P*-value < 0.001), and BMI for age Z-score (*P*-value < 0.001). Moreover, STRONGkids was found to be associated with weight for height Z-score (*P*-value: 0.017).

**Conclusion:**

The PYMS is a practical and beneficial tool in early identifying the risk of severe malnutrition in hospitalized patients. It is a suitable method for patients in our settings.

**Supplementary Information:**

The online version contains supplementary material available at 10.1186/s40795-022-00525-8.

## Background

Malnutrition in children is considered to be a determining factor of mortality and morbidity, especially in low- and middle-income areas. Malnutrition can cause serious financial and social problems in high-risk groups and thus impose a heavy economic burden on countries [1]. Malnutrition is associated with underdevelopment of the brain and leads to cognitive and intellectual disabilities [2]. It appears that hospitalized children are at a higher risk of malnutrition, which can affect the length of stay (LOS) in hospital and mortality [3, 4]. The importance of prompt diagnosis and management of malnutrition has been repeatedly proven and different malnutrition screening tools have been proposed for use in children and adults [5]. Weight for height and Body Mass Index (BMI) for age Z-score are worldwide accepted tools to detect malnutrition. However, the anthropometric measures cannot identify children in the early stages of malnutrition or those at risk of worsening due to an acute medical condition [6]. That is why clinicians have tried to find more suitable screening tools for malnutrition. To date, there is no consensus on the ideal screening tool for the early diagnosis of malnutrition in hospitalized children [7]. Among the many screening tools proposed for children, none is routinely used clinically. Many of these tools are simple to apply and greatly beneficial [8] various methods are currently used including:

1. Screening Tool for the Assessment of Malnutrition (STAMP) was designed and validated in 2007 in the U.K for children of 2 to 17 years [9, 10].
2. Pediatric Yorkhill Malnutrition Score (PYMS) introduced in 2008 [11].
3. Screening Tool for Risk on Nutritional Status and Growth (STRONGkids), validated in 2007 in The Netherlands [12].

These three screening tools have been used in outpatient and hospital settings and many studies have been conducted to investigate their sensitivity and specificity [9]. These tools consider patients’ recent food intake, medical condition, or the anticipated change in the near future in the diet. These are the reasons making the mentioned screening tools invaluable and distinct from conventional tools for assessing malnutrition.

Due to the importance of nutrition in hospitalized patients, discovering the ones susceptible to malnutrition could lead to early intervention and as a result, shorter stay in hospital and less morbidity. No study has yet tried to identify and analyze the patients at risk for malnutrition in our area. We decided to combine anthropometric indices and screening tools of STAMP, PYMS and STRONGkids. This association could inform us about individual nutritional requirements that need attention and intervention and the prevalence of malnutrition in admitted patients. By acknowledging the magnitude of the problem, we will be able to provide strategies for attending patients at-risk or affected by malnutrition. Besides, comparing the three malnutrition screening tools with conventional methods could yield in finding the ability of each one in discovering at risk patients.

## Methods

### Study design

In a cross-sectional study, children from 1 to 16 years old who were admitted for more than 48 h in infectious, general, and surgery wards of a tertiary pediatrics hospital, Bahrami Children Hospital, Tehran, (Iran) in a 2-month period were included. All the patients admitted in the mentioned wards were included, literally all the patients admitted in each day, with different reasons for admission, were included (except for the exclusion criteria). Reasons for admission included gastroenteritis, urinary tract infections and pneumonia in infectious ward, diseases of GI tract and renal diseases in general ward, and patients admitted for surgeries like appendicitis and genitourinary abnormalities in surgery ward. Patients with immunodeficiencies and malignancies and patients who were admitted for less than 48 h were excluded. Informed consent was obtained from the patients’ parents upon admission. The aim of the study and the methods were explained well to the parents, and all of the parents were willing to participate. Height and weight were measured when patients were admitted and discharged by a trained nurse. The height of the children less than 2 years old was measured with a meter while lying down and the ones older than 2 were measured while standing up. Weight for children less than 2 years old was measured by the infants’ scale. The valid questionnaires of STRONGkids, STAMP and PYMS were filled for each patient by a pediatrics resident, who had been taught by studying the manuals of each risk score. The questionnaires can be found in the Supplementary Tables 1, 2, and 3. In STRONGkids, the nutritional status was surveyed considering clinical symptoms, underlying disease, changes in nutritional intake and output, and changes in weight. There was four questions in this questionnaire, and the total scores can be 0 to 5, which scores 4 and 5 refer to high malnutrition. STAMP evaluated underlying disease, nutritional intake and differences in weight and height percentiles, STAMP has three questions and the total score varies from 0 to 9. The score 4 and higher means a high risk of malnutrition. PYMS analyzed weight loss, BMI, nutritional intake changes and the possibility of changing the patient's food intake in the future. It comprises of four questions, with total score of 0 to 7. The total score of 2 and higher stands for high risk of malnutrition. The prevalence of malnutrition in each ward during the research time was estimated.

The world health organization (WHO) and Centers for Disease Control and Prevention (CDC) growth charts were respectively used to determine the nutritional status in children under 2 years of age and over 2 years of age, as recommended by the American Academy of Pediatrics (AAP) and the CDC [13]. The anthropometric measurements of each child was plotted on weight for height and BMI for age charts. Based on the Z-score lines provided by the weight for height charts, Z-score less than -3 was considered as “severe malnutrition”, -3 to -2 as “moderate”, -2 to + 2 as “normal”, and + 2 to + 3 as “overweight. Based on the Z-score lines provided by the BMI for age charts, Z-score less than -3 was grouped as “severely wasted”, -3 to -2 as “moderately wasted”, -2 to + 1 as “normal”, + 1 to + 2 as “overweight” and higher than + 3 as “obese” [14].

The following formula was used to estimate the sample size, where P was considered as 0.32 based on a study by Spagnuolo et al. [15]. Assuming a significance level of 5% and an effect size of 10%, the sample size was calculated as 83.

$$n=\frac{{Z}_{1-\frac{a}{2}}^{2}p\left(1-p\right)}{{d}^{2}}$$

*n*= (1.96)^2^ *0.32 *0.68 / (0.1)^2^ = 83

### Ethical consideration

The study adhered to the tenets of the Helsinki Declaration. All the information was kept confidential. No changes were applied to the treatment strategies due to this study. No additional costs were imposed on the family and no intervention was performed on the patient. Informed consent was obtained to participate from parents of minors. This study was approved by the ethics committee of Tehran University of Medical Sciences, Children's Medical Center, and the code of ethics was assigned to it: IR.TUMS.CHMC.REC.1397.092.

### Statistical analysis

We analyzed the data by SPSS, 25^th^ version. The descriptive data was reported by descriptive statistics (mean, standard deviation, frequency, relative frequency). Chi square test and Fisher’s exact test were used to compare qualitative variables. For comparing quantitative variables, the student t-test was used for normal distribution and Mann–Whitney for non-normal. Pearson’s correlation coefficient was used to determine correlation. The *P*-Values less than 0.05 were considered significant. ANOVA test was used to analyze significant difference in malnutrition rates in the different wards. Sensitivity, specificity, positive predictive value, and negative predictive values of the risk scores based on weight for height and BMI for age Z-scores were calculated by crosstabulation.

## Results

A total of 93 patients were enrolled in this study, with a mean age of 5.53 ± 3.9 years, the mean height of 112 ± 20 cm, mean weight of 22.6 ± 14.4 kg and mean BMI of 16.58 ± 4.31 kg/m^2. The demographic data are summarized in Table 1.

**Table 1:** Demographic data of the patients

Variables	Mean ± Standard Deviation	Range
Weight (kg)	22.7 ± 14.4	6–73
Height (m)	1.1 ± 0.2	0.6- 1.6
BMI (kg/m^2^)	16.6 ± 4.3	9.7–32.4
Age (years)	5.5 ± 3.9	1–16
Length of stay (days)	5.2 ± 1.5	5.2–10
Weight in admission (kg)	22.7 ± 14.3	22.7–68

Sixteen percent of the patients were admitted at the surgery ward, 41% at the general ward, and 43% at the infectious diseases ward. Patients’ data according to the admitted ward are shown in Table 2. The reasons for admission are summarized in the supplementary Table 4.

**Table 2:** Demographic data according to admitted ward

	Wards	No	Mean ± SD	Range
Weight (kg)	General	37	24.52 ± 15.90	6–61
	Infectious	39	20.74 ± 13.95	8–73
	Surgery	15	23.23 ± 11.91	10–55
Height (m)	General	37	01.14 ± 00.25	0.7–1.6
	Infectious	38	01.09 ± 00.25	0.7–1.5
	Surgery	14	01.14 ± 00.24	0.7–1.4
BMI (kg/m^2^)	General	37	16.76 ± 04.24	11.6–30
	Infectious	38	16.00 ± 04.30	9.7–32.4
	Surgery	14	17.65 ± 04.57	12.3–27.6
Age (yr.)	General	37	05.89 ± 04.42	1–16
	Infectious	39	04.98 ± 03.56	1–13
	Surgery	15	06.06 ± 03.28	1–10

According to the weight for height Z-score, 66% of the patients were categorized as “normal”, 19% as “moderate malnutrition”, 7% as “severe malnutrition”, 2% as “overweight” and 6% as “obese”. BMI for age divided patients as 34% “normal”, 17% “moderately wasted”, 22% “severely wasted”, 12% “overweight” and 15% “obese”. No significant difference could be found in malnutrition prevalence between different wards by ANOVA test.

PYMS scores ranged from 0 to 5, median 4:; STRONGkids scores ranged from 0 to 5, median:2, and STAMP scores ranged from 0 to 8, median:4.

We found a significant relationship between PYMS and weight for height Z-score (*P*-value < 0.001). It should be noted, though, that weight for height Z-score is only provided for children under 6 years old by CDC and WHO, and that is why a total of 53 patients were included in this analysis. Moreover, the STRONGkids score was significantly associated with weight for height Z-score when performing Fisher’s Exact Test (*P*-value = 0.02). STRONGkids also revealed a relatively good ability to detect low-risk patients (79.5% or 31 patients) but performed poorly for high-risks (25% or 1 patient). STAMP did not show such significant relationships (*P*-value = 0.76).

PYMS showed a significant relationship with the BMI for age Z-score (*P*-value < 0.001) while the two other risk scores were not significantly related to the BMI for age Z-score. Relationships between PYMS and STRONGkids and weight for height z-score showed in the Table 3, and relationship between PYMS and BMI for age in the Table 4.

**Table 3:** Relationship between PYMS and weight for height Z-score by Pearson Chi Square in patients less than 6 years old (*N* = 53)

		Weight for height value	Weight for height value	Weight for height value		
		Severe malnutrition	Moderate malnutrition	Not malnourished	Total	*P*-value
PYMS	Low-risk	0 (0.0%)	0 (0.0%)	25 (64.1%)	25 (47.2%)	
	Moderate risk	0 (0.0%)	2 (20.0%)	5 (12.8%)	7 (13.2%)	< 0.001
	High-risk	4 (100.0%)	8 (80.0%)	9 (23.1%)	21 (39.6%)	
**Total**		4 (100.0%)	10 (100.0%)	39 (100.0%)	53 (100.0%)	
STRONG	Low-risk	0 (0.0%)	5 (27.8%)	31 (40.2%)	36 (35.2%)	
kids	Moderate risk	3 (42.8%)	3 (16.6%)	7 (9.0%)	13 (12.7%)	0.017
	High-risk	1 (25.0%)	2 (20.0%)	1 (2.5%)	4 (7.5%)	
**Total**		4 (100.0%)	10 (100.0%)	39 (100.0%)	53 (100.0%)	

Table 3 depicts the number of patients in crosstabs in subgroups of weight for height Z score and PYMS. After cross tabulation, *P*-value for Chi Square and Fisher’s Exact Test analysis are given

**Table 4:** Relationship between PYMS and BMI for age Z-score by Pearson Chi Square in all the studied patients (*N* = 93)

	BMI for age value					
		Severe malnutrition	Moderate malnutrition	Not malnourished	Total	*P*-value
PYMS	Low-risk	2 (10.0%)	6 (50.0%)	36 (63.2%)	44 (49.4%)	
	Moderate risk	2 (10.0%)	3 (25.0%)	14 (24.6%)	19 (21.3%)	< 0.001
	High-risk	16 (80.0%)	3 (25.0%)	7 (12.3%)	26 (29.2%)	
Total		20 (100.0%)	12 (100.0%)	57 (100.0%)	89 (100.0%)	

Table 4 depicts the number of patients in crosstabs in subgroups of BMI for age Z score and PYMS. After cross tabulation, *P* value for Chi Square analysis is given

Sensitivity, specificity, positive predictive value, and negative predictive value of the risk scores based on weight for height and BMI for age Z-scores are summarized in Table 5.

**Table 5:** Sensitivity, specificity, positive predictive value, and negative predictive values of the risk scores based on weight for height and BMI for age Z-scores

Risk Score	Category	Sensitivity		Specificity		PPV		NPV	
		BMI for age	Weight for height	BMI for age	Weight for height	BMI for age	Weight for height	BMI for age	Weight for height
STAMP	Low-risk	19%	23%	81%	93%	65%	90%	36%	30%
	High-risk	90%	100%	35%	26%	28%	10%	92%	100%
PYMS	Low-risk	63%	64%	75%	100%	81%	100%	53%	50%
	High-risk	80%	100%	85%	65%	61%	19%	94%	100%
STRONGkids	Low-risk	68%	79%	44%	64%	68%	86%	58%	53%
	High-risk	20%	25%	29%	94%	50%	25%	25%	94%

Data are presented as relative frequencies; *NPV* Negative Predictive Value, *PPV* Positive Predictive Value, *PYMS* Paediatric Yorkhill Malnutrition Score, *STRONGkids* Screening Tool Risk on Nutritional status and Growth, *STAMP* Screening Tool for the Assessment of Malnutrition in Paediatric

The median for LOS was 5 days (IQR 4 to 6) and it was shown to be associated with STRONGkids score (*P*-value = 0.050), and was not associated with the two other scores, BMI for age or weight for height Z-scores.

## Discussion

An appropriate nutritional status can have a critical role in normal growth and development, response to treatment, ameliorating the quality of life, lessening admission expenses and increasing survival [8, 16].

According to the Weight for height Z-score in our study, a total of 26.42% of children were malnourished (moderate or severe), and according to BMI for age Z-score, 39.32% were wasted. Different studies over the past decade have reported malnutrition prevalence of 5.1% to 55.6%. [17]. The prevalence was reported as 48.5% in rural Ethiopia in 2017 [18]. Baxter et al. declared 8.51% malnutrition prevalence in a hospital in Canada in 2014 [19]. In 2018, Dehghani et al. reported the prevalence of malnutrition in children without previous admission history as 48.5% in Shiraz, Iran [20]. Mahdavi et al. in 2008 in Tabriz, Iran, reported malnutrition prevalence according to weight for age, height for age, weight for height, and skinfold thickness on triceps as 48.6%, 30.7%, 32.2% and 14.3%, accordingly [21]. Our reported measures are still considerably high compared to previous studies conducted in Iran. Differences in these measurements could be attributed to improvements in parents’ knowledge about nutritional needs and differences in assessment tools and study locations.

STAMP, STRONGkids, and PYMS are valid questionnaires used as screening tools for malnutrition in different world regions [22–25]. The current study found a significant relationship between PYMS and STRONGkids and Weight for height Z-score and also with PYMS and BMI for age. Kastagoni et al. surveyed 1,506 children in two Greek hospitals and compared the efficacy of PYMS and STAMP screening tools. They assessed the agreement of these tools with dietitians’ judgments, and reported that PYMS showed better yet moderate agreement with the dietitians’ judgment (kPYMS_WHO = 0.47; 95%CI 0.41–0.52) compared to STAMP (kSTAMP_WHO = 0.28; 95%CI 0.23–0.33) [26]. Hulst et al. performed a multi-center study in 44 hospitals and STRONGkids. They found a significant association between having a "high risk" score of STRONGkids and a negative SD-score in weight-for-height [12]. PYMS and STRONGkids can detect malnutrition in earlier stages compared with anthropometric measures, and they attend to patients’ nutrition changes due to hospitalization; that is why they can be useful tools applied to hospitalized children. Considering their helpfulness in case detection and convenience of use, PYMS and STRONGkids can be good candidates for early detection of malnutrition in admitted patients in our settings. A nutrition consult for these patients can reduce complications of malnutrition and improve health indices [27]. Since the identification of high-risk cases of malnutrition is essential for preventing severe malnutrition, STAMP is not assumed to be beneficial; yet PYMS was proved to be potent in identifying high-risk patients by Weight for height Z-score in almost all the cases and by BMI for age with 80% potency. Regarding low-risk patients, PYMS can detect more than 60%. Although the European Society for Pediatric Gastroenterology, Hepatology and Nutrition has recommended screening for malnutrition, there are no standard routine screening tools for hospitalized children [28]. This study can depict a regional standard for screening the nutritional status of hospitalized children. Table 5 shows sensitivity, specificity, PPV and NPV of each screening tool based on BMI for age and weight for height Z score. PYMS shows a reasonably higher sensitivity and specificity both is low risk and high-risk groups, compared to the other two screening tools. The study of Pars et al. in 2020 in Turkey yielded the same result, as PYMS showed higher overall sensitivity and specificity in comparison with STRONGkids and STAMP [29].

Only a few studies have discussed or implemented nutrition screen tools in Iran. Khajavi et al. used STRONGkids to assess the nutritional status of pediatric cancer patients in 2020 [30]. Moeeni et al. recruited 119 patients in a study in order to assess the three nutritional risk scores. They concluded that STRONGkids correlated more strongly with anthropometric measures [31]. Imani et al. applied STAMP, PYMS, and STRONGkids in a group of hospitalized children and tried to determine their validity. They reported that PYMS was able to detect a higher number of malnourished children according to anthropometric measures [32]. The current study can be used as a benchmark for other Iranian studies since it provides a detailed analysis of the three screening tools.

An important feature of a screening score is the ability to predict clinical values such as length of stay in the hospital. According to our study, only the STAMP score was correlated with LOS. We could not find such a correlation between LOS and the two other scores, weight for height Z-score or BMI for age Z-score. In the study of Chourdakis et al. conducted in 2010–2011 on 2567 patients of twelve European countries, it was shown that children at high risk for the three risk scores (STRONGKIDS: 10%, PYMS: 25%; and STAMP: 23%) had a longer LOS than that of children at low risk [33].

Regarding the efficacy of PYMS and STRONGkids use in children, we propose that children with moderate and high scores of PYMS and STRONGkids be referred to nutritionists. There is a gap in the literature in assessing the role of intervention by nutritionists in such groups; we suggest that more investigations are done in this area to help determine the use of PYMS and STRONGkids practically and permitting the children in developing countries to benefit from it.

## Conclusion

Malnutrition is a determining factor of pediatric mortality and morbidity, especially in low- and middle-income countries. Hospitalized children are at a higher risk of malnutrition. The PYMS and STRONGkids are practical and beneficial tools in early identifying the risk of severe malnutrition in hospitalized patients. They are suitable methods for patients in our settings.

### Recommendation

Future studies can be done with larger populations. We believe stronger correlations can be found between PYMS and STRONGkids and Z scores in larger studies. Also, now that we know these screening tools can be a reasonable tools for detecting malnutrition, studies can be done to validate this questionnaire for the use of Iranians, and also for assessing the ease of use of this tool in hospitals.

## Supplementary Information

**Additional file 1: Supplementary Table 1.** STRONGkids tool. **Supplementary Table 2. **PYMS tool. **Supplementary Table 3.** STAMP tool. **Supplementary Table 4.** A summary of reasons for admission.

## Data Availability

The datasets used and/or analyzed during the current study are available from the corresponding author on reasonable request.

## Abbreviations

ANOVA: Analysis of variance
BMI: Body Mass Index
IQR: Interquartile range
LOS: Length of stay
NPV: Negative Predictive Value
PPV: Positive Predictive Value
PYMS: Pediatric Yorkhill Malnutrition Score
STAMP: Screening Tool for the Assessment of Malnutrition
STRONGkids: Screening Tool for Risk on Nutritional Status and Growth
