# Supplementary material for: Evaluation of the risk of malnutrition in hospitalized children by PYMS, STAMP, and STRONGkids tools and comparison with their anthropometric indices: a cross-sectional study
Source: BMC Nutr. 2022 Apr 21;8:33. doi: 10.1186/s40795-022-00525-8 (PMC9027700; doi:10.1186/s40795-022-00525-8)
Supplement: Supplementary file 1 — Additional file 1: Supplementary Table 1. STRONGkids tool. Supplementary Table 2. PYMS tool. Supplementary Table 3. STAMP tool. Supplementary Table 4. A summary of reasons for admission. [file 40795_2022_525_MOESM1_ESM.docx]

**Supplementary Table 1: STRONGkids tool**

|  | **STRONGkids** | **score** |
| --- | --- | --- |
| 1 | Is there an underlying illness with risk for malnutrition (see list) or expected major surgery? | 1 |
| 2 | Is the patient in a poor nutritional status judged with subjective clinical assessment: loss of subcutaneous fat and/or loss of muscle mass and/or hollow face? | 2 |
| 3 | Is one of the following items present?   - Excessive diarrhea (≥5 per day) and/ or vomiting (> 3 times/ day) during the last 1-3 days - Reduced food intake during the last 1-3 days - Pre-existing nutritional intervention (e.g. ONS or tube feeding) - Inability to consume adequate nutritional intake because of pain | 1 |
| 4 | Is there weight loss (all ages) and/or no increase in weight/height (infants < 1year) during the last few week-months | 1 |
|  | **Total Score** |  |

Hulst JM, Zwart H, Hop WC, Joosten KF. Dutch national survey to test the STRONGkids nutritional risk screening tool in hospitalized children. Clin Nutr. 2010;29(1):106-11.

| **Diseases with risk of malnutrition (item1)** | |
| --- | --- |
| Psychiatric eating disorder  Burns  Bronchopulmonary dysplasia (up to age 2 years)  Celiac disease (active)  Cystic fibrosis  Dysmaturity/prematurity (until corrected age 6 months)  Cardiac disease, chronic  Infectious disease  Inflammatory bowel disease  Cancer | Liver disease, chronic  Kidney disease, chronic  Pancreatitis  Short bowel syndrome  Muscle disease  Metabolic disease  Trauma  Mental handicap/retardation  Expected major surgery  Not specified (classified by doctor) |

**Supplementary Table 2: PYMS tool**

|  | **PYMS** | **Yes/No** | **Score** |
| --- | --- | --- | --- |
| 1 | Is the BMI below the cut-off value in the table overleaf? | No | 0 |
|  |  | Yes | 2 |
| 2 | Is the patient in a poor nutritional status judged with subjective clinical assessment: loss of subcutaneous fat and/or loss of muscle mass and/or hollow face? | No | 0 |
|  |  | Yes | 1 |
| 3 | Has the child had a reduced intake (including feeds) for at least the past week? | No | 0 |
|  |  | Yes (Decreased) | 1 |
|  |  | Yes (No intake or very little) | 2 |
| 4 | Will the child’s nutrition be affected by the recent admission/condition for at least the next week? | No | 1 |
|  |  | Yes | 2 |
|  | **Total Score** | |  |

Gerasimidis K, Keane O, Macleod I, Flynn DM, Wright CM. A four-stage evaluation of the Paediatric Yorkhill Malnutrition Score in a tertiary paediatric hospital and a district general hospital. Br J Nutr. 2010;104(5):751-6.

**Supplementary Table 3: STAMP tool**

|  | **STAMP** | **Yes/No** | **Score** |
| --- | --- | --- | --- |
| 1 | Does the child have a diagnosis that has any nutritional implications? | Definitely | 3 |
|  |  | Possibly | 2 |
|  |  | No | 0 |
| 2 | What is the child’s nutritional intake? | None | 3 |
|  |  | Recently decreased/none | 2 |
|  |  | No change/good | 0 |
| 3 | Use a growth chart or the centile quick reference tables to determine the child’s measurements | > 3 centile spaces/≥ 3 columns apart (or weight < 2nd centile) | 3 |
|  |  | >2 centile spaces/=2 columns apart | 1 |
|  |  | 0 to 1 centile spaces/columns apart | 0 |
|  | **Total Score** |  |  |

Wong S, Graham A, Hirani SP, Grimble G, Forbes A. Validation of the Screening Tool for the Assessment of Malnutrition in Paediatrics (STAMP) in patients with spinal cord injuries (SCIs).

**Supplementary Table 4: A summary of reasons for admission**

| **The diagnosis upon admission** | **Number of cases** |
| --- | --- |
| Arthritis | 3 |
| Respiratory distress/pneumonia | 19 |
| Kawasaki | 2 |
| Metabolic disease | 5 |
| Urologic surgery | 8 |
| Gastrointestinal Tract Surgery | 7 |
| ENT surgery | 2 |
| Febrile convulsion | 8 |
| Sinusitis | 2 |
| Diabetes hyperglycemia | 7 |
| Henoch Schoenlein Purpura | 1 |
| Gastroenteritis | 8 |
| GI bleeding | 2 |
| lymphadenitis | 2 |
| UTI | 4 |
| Nephrotic syndrome | 1 |
| Fever without localizing sign (FWLS) | 2 |
| ITP | 1 |
| Favism | 1 |
| Seizure | 2 |
| Abdominal pain workup | 1 |
| Chest pain | 1 |
| Herpangina | 1 |
| Retropharyngeal abscess | 1 |
| Meningitis | 1 |
| Bacteremia | 1 |
| **Total** | 93 |
